# Clinical and radiological outcomes of acetabular revision surgery with trabecular titanium cups in Paprosky type II and III bone defects

**DOI:** 10.1186/s10195-021-00571-1

**Published:** 2021-03-06

**Authors:** Loris Perticarini, Stefano Marco Paolo Rossi, Marta Medetti, Francesco Benazzo

**Affiliations:** 1 Sezione Di Chirurgia Protesica Ad Indirizzo Robotico - Unità Di Traumatologia Dello Sport, U.O. Ortopedia e Traumatologia Fondazione Poliambulanza, Via Bissolati 57, 25124 Brescia, Italy; 2 Clinica Ortopedica e Traumatologica, Fondazione IRCCS Policlinico San Matteo, Pavia, Italy; 3 Università Degli Studi Di Pavia, Pavia, Italy

**Keywords:** Revision total hip arthroplasty, Acetabular bone loss, Acetabular reconstruction, Trabecular titanium cup

## Abstract

**Background:**

This prospective study aims to evaluate the mid-term clinical outcomes and radiographic stability of two different types of cementless trabecular titanium acetabular components in total hip revision surgery.

**Methods:**

Between December 2008 and February 2017, 104 cup revisions were performed using trabecular titanium revision cups. Mean age of patients was 70 (range 29–90; SD 11) years. The majority of revisions were performed for aseptic loosening (86 cases, 82.69%), but in all the other diagnoses (18 cases), a significant bone loss (Paprosky type II or III) was registered preoperatively. Bone defects were classified according to Paprosky acetabular classification. We observed 53 type II defects and 42 type III defects. Cups were chosen according to the type of defect.

**Results:**

Average follow-up was 91 (range 24–146) months. Mean Harris Hip Score (HHS) improved from 43.7 (range 25–70; SD 9) preoperatively to 84.4 (range 46–99; SD 7.56) at last follow-up. One (1.05%) cup showed radiographic radiolucent lines inferior to 2 mm and was clinically asymptomatic. One (1.05%) cup was loose and showed periacetabular allograft reabsorption. Kaplan–Meier survivorship was assessed to be 88.54% (95% CI 80.18–93.52%) at 71 months, with failure of the cup for any reason as the endpoint.

**Conclusion:**

Trabecular titanium revision cups showed good clinical and radiographic results at mid-term follow-up in Paprosky type II and III bone defects.

**Level of evidence:**

Level IV prospective case series

## Background

Mechanical loosening is one of the most common indications for revision in total hip arthroplasty (THA) in the United States, as reported by Bozic et al. [1]

Main issue of cementless acetabular fixation is to guarantee a stable primary fixation to favor osteointegration and its long-term survivorship. Porous materials had been developed to provide a support for the biological fixation to the bone [2]. Different kinds of coating or 3D structures are used to improve capability of a primary fixation and secondary osteointegration. Trabecular Metal™ derived from Tantalum was the first porous material extensively used in hip (and knee) revision surgery with promising results [3–5]. Subsequently, different manufacturers developed different porous materials or coatings with different technologies [6]. Trabecular titanium (TT) is a highly porous solid structure characterized by a regular three-dimensional hexagonal-cell structure, which is designed to imitate the morphology of the trabecular bone [7, 8].

Recent in vitro studies demonstrated that trabecular titanium has osteoconductive proprieties and can contribute to create an osteoinductive environment stimulating osteoblasts’ proliferation and differentiation [9–11]. In vivo histological and histomorphometric analysis in a sheep model comparing trabecular titanium with traditional porous coatings showed that trabecular titanium provides an optimal microenvironment to favor direct osteogenesis with high osseointegration. [12]

This study aims to evaluate the mid-term clinical and radiographic outcomes of cup revision procedures performed with trabecular titanium hip revision cups.

## Methods

Between December 2008 and February 2017, 104 cup revisions were performed using trabecular titanium revision cups. All operated patients, despite their diagnosis, had a significant bone loss.

The majority of revisions were performed for aseptic loosening (86 cases, 82.69%), but in all other diagnoses (18 cases, 17,31%), a significant bone loss (Paprosky type II or III) was registered preoperatively. Causes for the other 18 revisions were metallosis in eight (7.69%) cases, traumatic acetabular periprosthetic fractures with unstable component in four (3.86%) cases, acetabular cup rupture in two (1.92%) cases, recurrent dislocations in two (1.92%) cases, two-stage revision for infection in one (0.96%) case, and liner breakage in one (0.96%) case.

A total of 21 patients had two or more surgical procedures on their hips. Mean age of patients at time of revision surgery was 70 (range 29–90; SD 11) years. Overall, there were 69 (66.34%) females and 35 (33.66%) males. Mean body mass index (BMI) at time of surgery was 25.68 (range 17–36.67; SD 3.71).

Overall, nine patients were lost at follow-up. The remaining 95 patients were available for evaluation.

In total, 95 cups (sizes 46–66) were implanted, and fixation was enhanced by means of 2–6 screws in all cases to ensure optimal primary stability.

The Delta TT revision system (Lima-Corporate, San Daniele del Friuli, Italy) presents two different types of trabecular titanium acetabular cup, Delta Revision TT and Delta One TT. Both cups present multiple holes in the cranial area and an inferior reduced rim to improve ROM. The system presents internal modularity options (“face changers”) which include standard or protruded liners (+ 5 mm offset), with straight or angled (10°–20°) spacers. Modular liners are helpful to optimize acetabular offset, can enhance femoral head coverage, and can change the acetabular version.

**Fig. 1:**
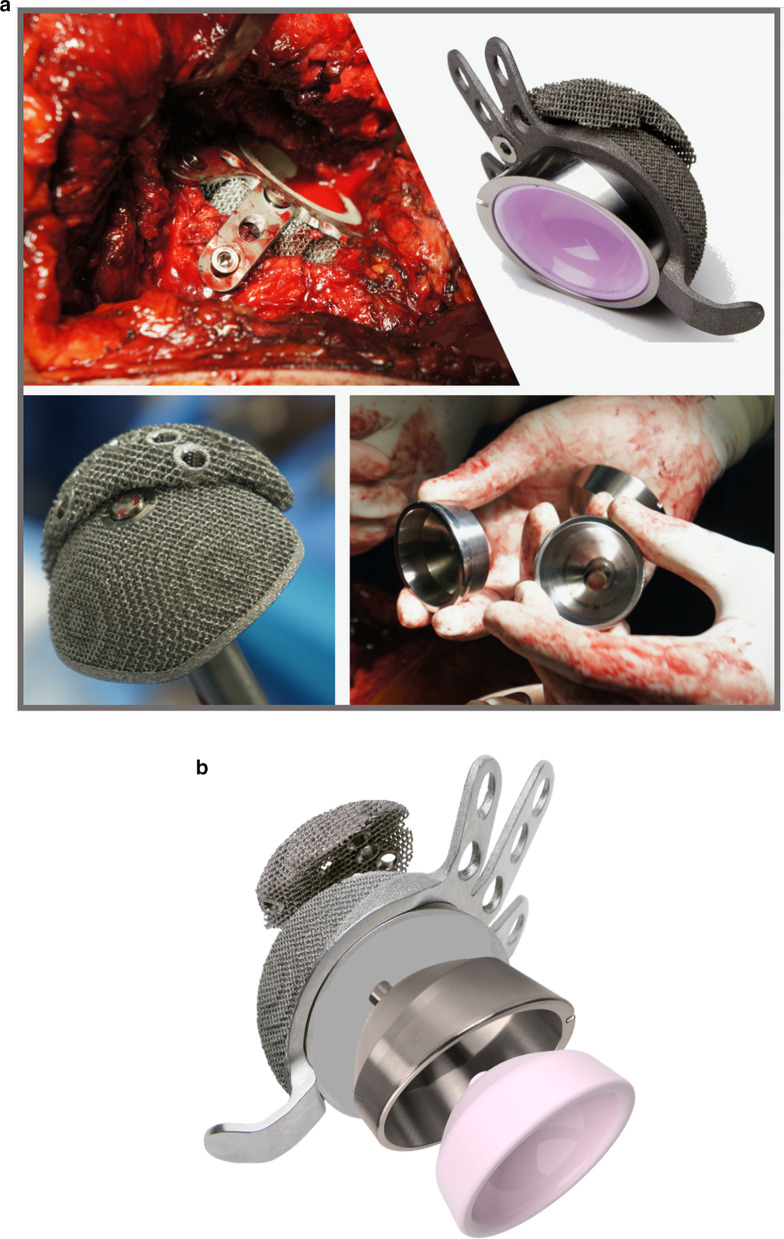
**a** From top left to bottom right: Intraoperative finding of a IIIB Paprosky acetabular defect treated with Delta TT Revision cup and TT augment fixed with screws on the cranial fins; Delta TT revision Cup with augment and face changer useful to lateralize the hip rotation center by 5 mm and improve the inclination cup angle by 20°; Delta One TT with polar augment already fixed; trial face changers. **b** A detail of the modularity of the cups and how to assemble the different components

The system also allows external modularity, represented by TT hemispherical augments. These are used to fill bone defects, increasing osseointegration. The Delta Revision TT cup is also characterized by a caudal hook for the obturatory foramen and three cranial fins for multiple screw placement options. Available bearings are ceramic and metal polyethylene liners. If the cup implanted is more than 50 mm, it is possible to use a metal insert and polyethylene mobile liner (Fig. 1a, b). A total of 39 (41.1%) patients received Delta Revision TT cup, and 56 (58.9%) patients received the Delta One TT cup.

All patients preoperatively underwent an AP view of the pelvis, a lateral view of the hip, and a pelvis CT-scan. Choice of cup was performed according to Paprosky acetabular classification, considering also the intraoperative founding.

We observed 53 type II defects and 42 type III defects. Type IIA, IIB, and IIC, presenting intact anterior and posterior acetabular walls, were treated with Delta One TT, except four cases of Paprosky type IIC, in which the reconstruction of the medial wall was managed with bone impaction grafting, mesh, and Delta Revision TT to protect the bone graft.

All the Paprosky type IIIA–IIIB were treated with Delta Revision TT, apart from four cases of type IIIA and three cases of type IIIB, which were treated with Delta One TT after reconstruction of the bone defects with the bone impaction grafting technique/modular augment or after fixation with plate and screws of the posterior wall (one case). To summarize, we used Delta One for Paprosy type II defect and Delta Revision for type III, with a small number of exceptions (eight cases) where the choice was intraoperative after evaluation of the quality of the walls.

**Fig. 2:**
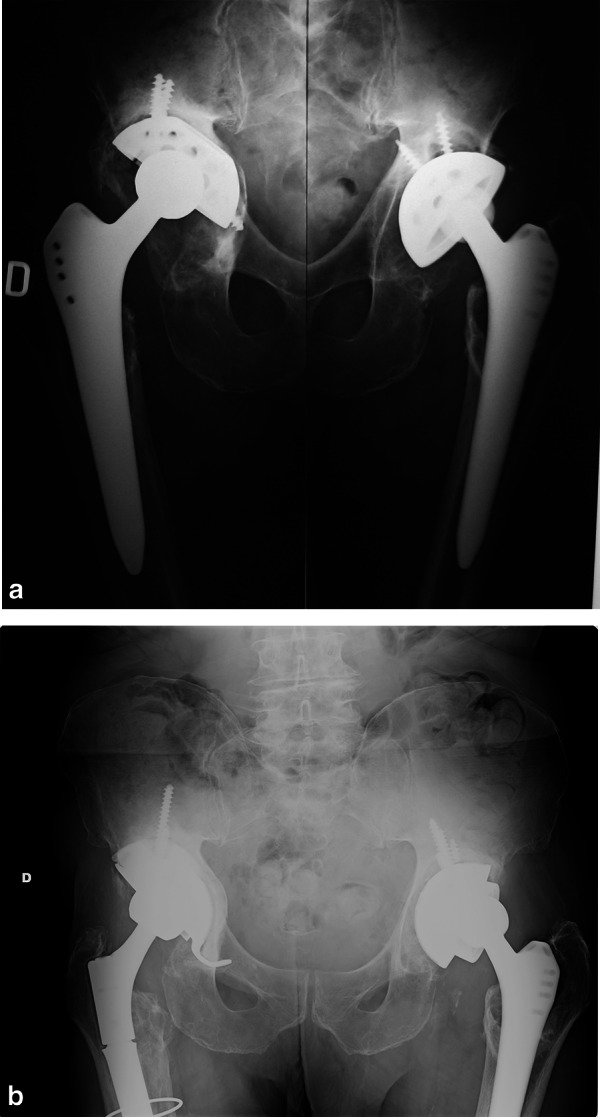
**a** Preoperative x-rays of bilateral cup aseptic loosening; **b** 9 years follow-up on right side with Delta TT Revision cup + augment and 8 years follow-up on left side using Delta One TT + face changer

**Fig. 3:**
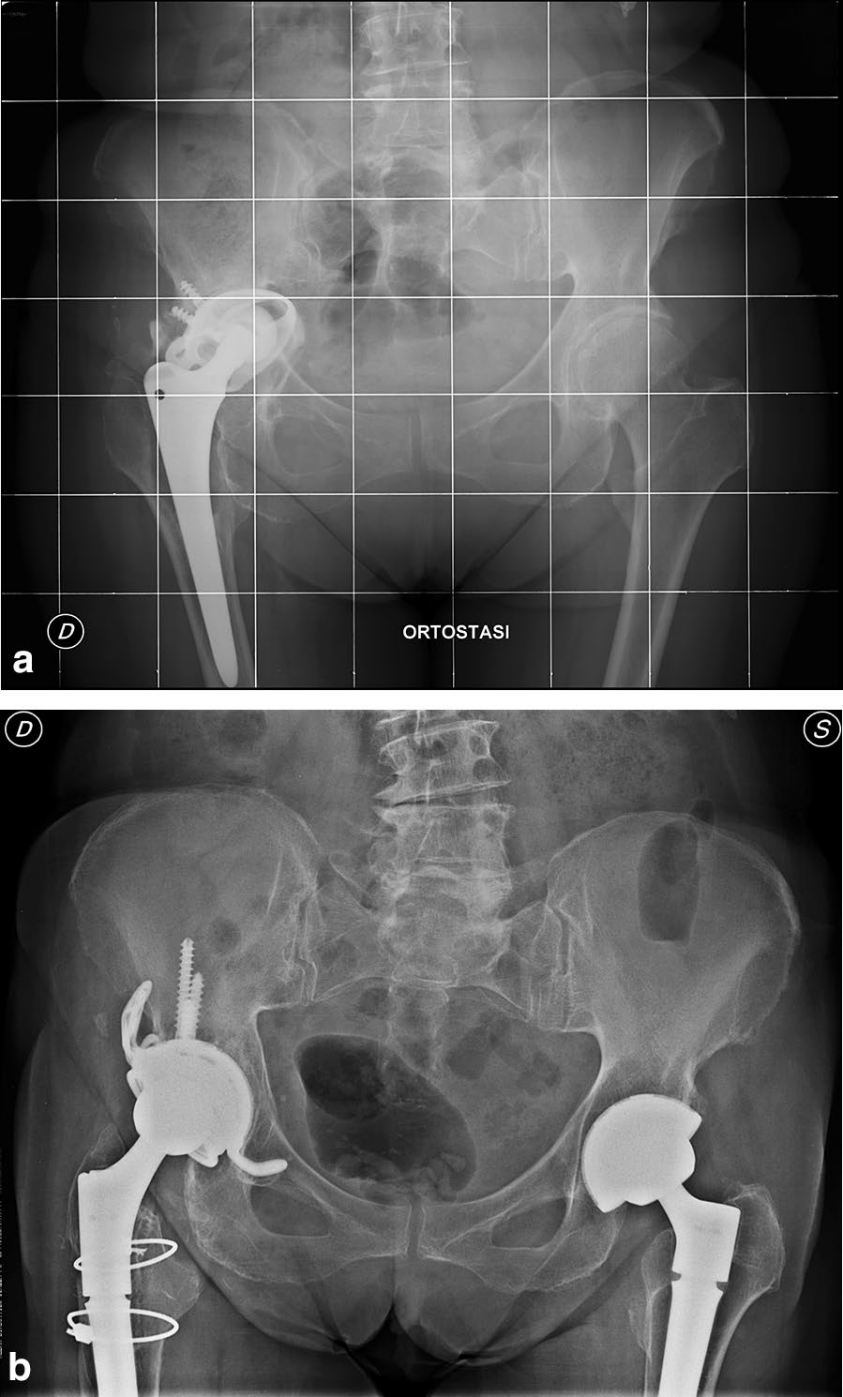
**a** Preoperative x-rays of cup loosening at right side; **b** 9 years follow-up after hip revision with Delta TT Revision cup, face changer, and modular stem on right side; 4 years follow-up after THA on left side

In 57 (60%) cases (31 Delta Revision TT and 26 Delta One TT), bone graft was used to fill cavitary defects (Table 1). Hemispheric TT augments were used in 13 cases with the same aim (Fig. 2a, b). Four meshes in Paprosky IIC were employed to support medial wall reconstruction. In one case, a trabecular metal (TM) augment, implanted in a previous revision surgery, was left in place and the cup was partially cemented. Face changers (or spacers) were used in 61 cases (Fig. 3a, b).

**Table 1:** Choice of implant and use of the bone according to Paprosky bone defects classification

	Paprosky classification				
	IIA	IIB	IIC	IIIA	IIIB
Delta one TT					
CUP	19	3	3	1	1 (After ORIF)
CUP + BIG	2	11	6	3	1
CUP + augment	1	1	\	\	\
CUP + augment + BIG	1	2	\	\	1
Delta revision TT					
CUP	\	\	\	2	5
CUP + BIG	\	\	4 (+ 4 Mesh)	12	9
CUP + augment	\	\	\	1	\
CUP + augment + BIG	\	\	\	3	3

to restore correct offset, cup coverage and version (Table 2).

**Table 2:** Combination of face changer, liner, dual mobility, and association to stem revision during the surgeries (*one patient changed only the neck of the modular stem)

	Neutral liner	Protruded liner	Dual mobility	Stem revision
Delta one TT				
No spacers	5	14	1	3*
Neutral	2	\	\	1
Protruded	9	9	\	5
Angled	4	3	\	2
Angled-protruded	5	2	2	1
Delta revision TT				
No spacers	3	10	1	7
Neutral	1	2	\	1
Protruded	\	5	1	2
Angled	\	5	\	2
Angled-protruded	4	6	1	1

Dual mobility was employed in six (6.3%) cases due to patient neurological poor condition.

Stem revision was also performed in 25 (26.3%) cases (Table 2); straight uncemented stems were used in 2 cases, a cemented stem in 1 case and modular conical primary and revision stems in 2 and 19 cases, respectively. Ten transfemoral osteotomies were performed to remove the stem. One patient underwent a change of the neck of the previous stem.

A posterolateral approach was used in all cases, with patients lying in a lateral decubitus position. All procedures were performed by a senior surgeon. In one (1.05%) case, a subcutaneous tenotomy of the adductor muscles was carried out to release a fixed contracture.

In one (1.05%) case of periprosthetic acetabular fracture, plate-and-screws fixation of the posterior column was carried out in the same surgical session. All patients were intravenously administered an antibiotic prophylaxis using 1 g of Vancomycin during surgery and 500 mg every 6 h until second day after surgery. Thromboembolic prophylaxis with low molecular weight heparin was administered postoperatively for a mean time of 6 weeks. Indomethacin (100 mg/day) was administered postoperatively for 30 days to prevent heterotopic ossifications.

A partial weight bearing with walking aids was allowed in cases treated with massive bone graft (Paprosky IIIA–IIIB) and femoral osteotomies, while other cases were granted a full weight bearing starting the first day after surgery. Early mobilization was encouraged after surgery.

Approval of our Institutional Review Board was obtained for this study, and all subjects provided informed consent prior to participation.

Clinical outcomes were evaluated using the Harris Hip Score (HHS) [13] before surgery, at immediate postoperative time, at 6 and 12 months after surgery, and once a year at follow-up subsequently. Radiographic evaluation was performed before surgery, at immediate postoperative time, at 6 and 12 months and subsequently once a year. Antero-posterior radiographs of the pelvis and lateral views of the affected hip were obtained to determine the cup inclination angle and to detect radiolucent lines, sclerosis, areas of osteolysis, and assess remodeling of the bone graft.

All measurements were made by a single observer. Patients were monitored in order to check for intra- and postoperative complications.

A Kaplan–Meier curve was performed to estimate the survivorship of TT Cups. Revisions of the acetabular component were considered as a failure.

Institutional review board approval for this study was obtained, with protocol number 20140001442.

## Results

The average follow-up was 91 (range 24–146) months. Five patients died after a mean time of 53 months after surgery (range 21–75) due to worsening of chronic pathological condition in three cases, and tumors in two cases, which were deemed as a cause unrelated to the surgical procedure.

Mean Harris Hip Score (HHS) improved from 43.7 (range 25–70; SD 9) preoperatively to 84.4 (range 46–99; SD 7.56) at last follow-up. We had no intraoperative complications.

Seven (7.3%) patients suffered of deep infection at a mean time of 35.85 months after surgery. Two of them had history of periprosthetic joint infection sustained by *Pseudomonas Aeruginosa and Staphylococcus Aureus*. All of them required two-stage revision surgery with good results at last follow-up. Seven (7.3%) patients underwent dislocation (two cases in Delta Revision TT series and five in Delta One TT series). Four (57.1%) of them were affected by neurological diseases.

Dislocations were treated by closed reduction in two cases, changing the modular component in two cases, while three patients required cup revision.

Two (2.1%) periprosthetic femoral shaft fractures occurred. One case was treated with open reduction and fixation with plate and screws, and the second case underwent a stem revision surgery using conical modular stem. We observed a single (1.05%) case of trochanteric bursitis.

**Fig. 4:**
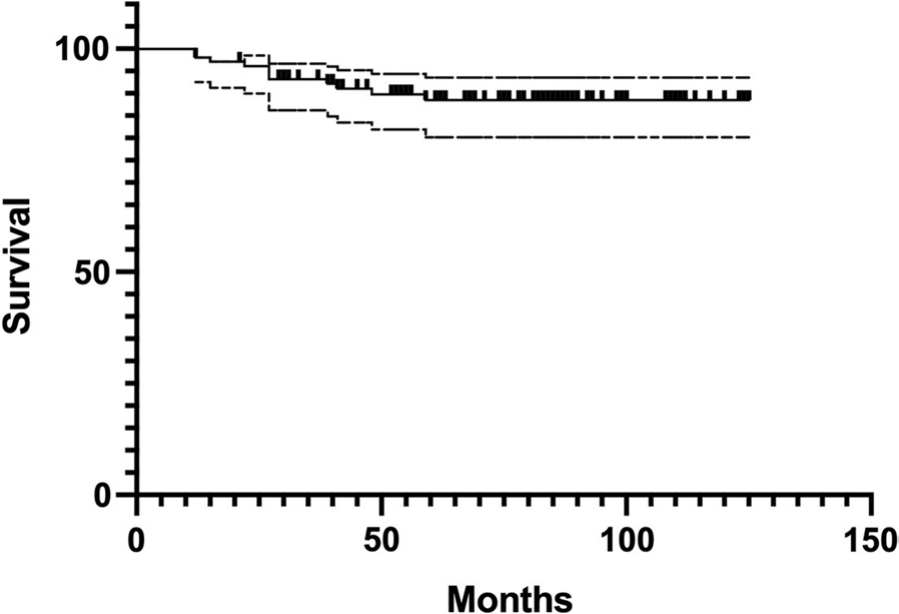
Kaplan–Meier survivorship curve, showing a survivorship of 88.54% (95% CI 80.18–93.52%) at 91 months of follow-up

Kaplan–Meier survivorship was assessed to be 88.54% (95% CI 80.18–93.52%) at 71 months, with failure of the cup for any reason as the endpoint (Fig. 4).

Postoperatively, a mean cup inclination at first radiological evaluation was 43.31° (range 26–64°; SD 7.15). In one (1.05%) case, the x-ray showed less than 2 mm radiolucent lines, which may have been indicative of aseptic loosening. Nonetheless, this case was clinically asymptomatic, the patient did not show any pain, limp, reduced range of motion, or signs of instability; thus, no revision surgery was performed.

There was one case of reabsorption of the graft, resulting in cup loosening 1 year after surgery (1.05%). It required a revision with a cage and cemented cup, showing good results at last clinical and radiological follow-up. Three cases of heterotopic ossification were observed (one case Brooker 1, two cases Brooker III), but they did not required surgery.

**Fig. 5:**
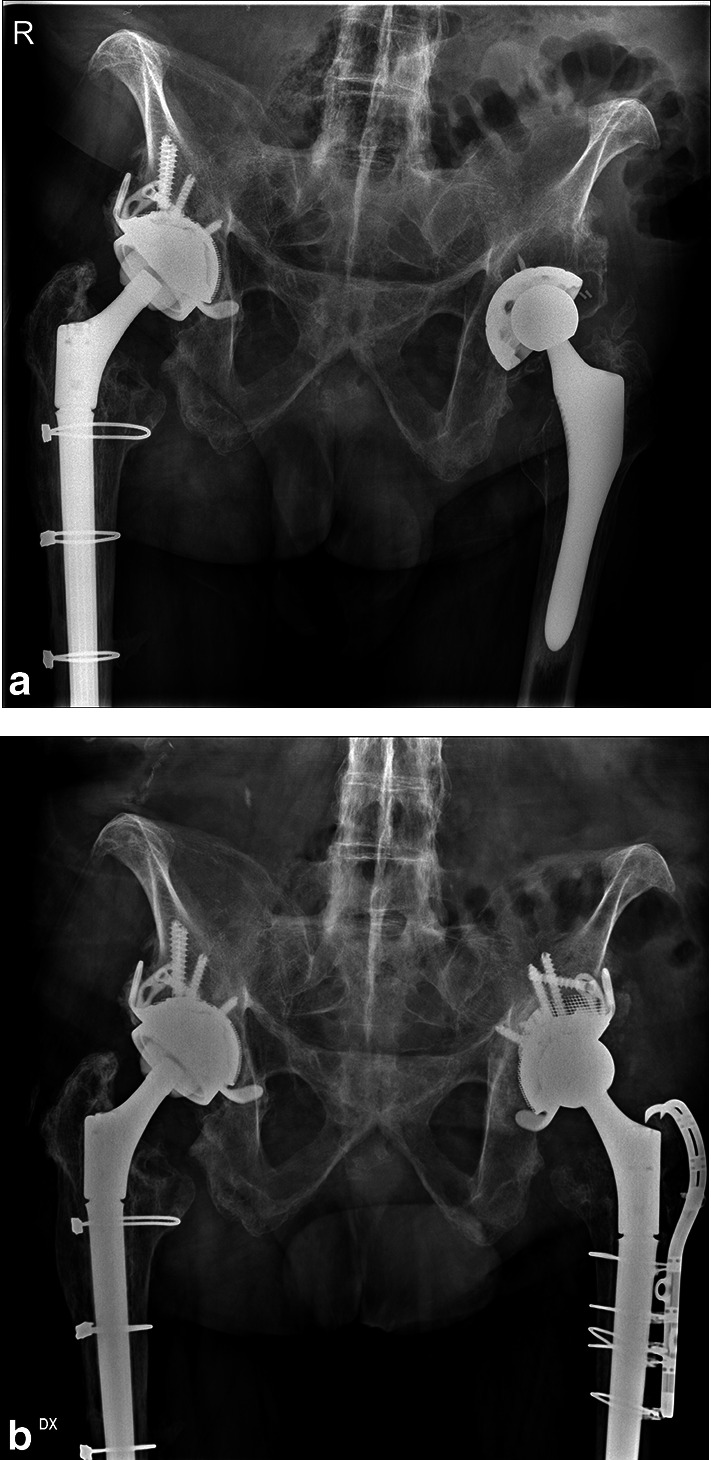
**a** On right side, Delta TT revision cup at 3 years of follow-up. On left side, preoperative x-rays showed cup loosening and bone defect; **b** 7 years follow-up on right side and 5 years follow-up on left side both with Delta TT revision cup, and augment and modular conical stems on left side

All other acetabular components were radiographically stable at last follow-up, showing evident signs of bone remodeling and osteointegration, without any radiolucent lines, sclerotic areas, or periprosthetic osteolysis (Fig. 5a, b).

## Discussion

This study aimed to evaluate the mid-term clinical and radiographic outcomes of cup revision procedures performed with trabecular titanium hip revision cups. The main finding of this report was that good clinical results were observed in our series, with a significant increase in patients’ quality of life. The mean Harris Hip Score improved from 43.7 (range 25–70) to 84.4 (range 46–99) at last follow-up, with a survivorship of 88.54% (95% CI 80.18–93.52%) at 91 months follow-up (maximum 146 months). To the best of the authors’ knowledge, this study presents the largest series and longer follow-up results of trabecular titanium revision implants. In the field of acetabular revisions, these results and survivorship can be considered good, as discussed by previous authors [14]. The main goal of the acetabular revision procedure is to fill the bone defects, obtain a good quality of reconstruction, and restore the correct center of rotation and muscle tension. Porous coatings and highly porous materials have been introduced to improve secondary stability of the implants to allow higher bone ingrowth [15]. These 3D porous structures have low modulus of elasticity and high frictional characteristics to achieve a better primary stability and mechanical performance. In recent years, in place of bulky allograft for acetabular loss, highly porous augments have been developed to achieve the restoration of the bone stock, leading to the reestablishment of center of rotation of the hip [16]. Our results are in line with recent papers reporting results with the use of trabecular metal (TM)-coated acetabular components that showed low risk of both aseptic and septic rerevision in cup revision surgery. These results are, in any case, comparable with those of non-TM components [4, 5, 17]. The main difference between trabecular titanium components and tantalum cups is the manufacturing system as they are produced with a 3D-printing technology known as electron beam melting (EBM), a high-energy focused beam used to locally melt metallic powders, layer upon layer, in a one-step manufacturing process. Structural continuity between the external highly porous trabecular surface and inner bulk is obtained. This provides higher structural solidity and tensile resistance, reducing risk of delamination and shedding typical of traditional porous coatings [18]. Trabecular titanium showed good results in difficult primary cases [19]. Our results are in line with those presented by De Meo et al. [20] in a recent report using trabecular titanium in hip revision with an overall survivorship of the cup of 94.8% at a shorter mean follow-up (48.3 months). The authors in their case series reported a rate of aseptic loosening of 1.5% at 48.3 months. Another study published by Gallart et al. [21] reported a 2.9% of aseptic loosening at 30.6 months of follow-up in acetabular revisions with TT components. Our results in terms of aseptic loosening (1.05%) are in line with those presented by both studies. Concerning our complications, we observed in our series seven cases of deep infection, which can be considered as late as they occurred at a mean 35.85 months after surgery, and they cannot be related to the procedures. We reported seven cases of dislocation in our series, which can be explained by the incidence of neurological diseases (57.1%) among patients, but also by the complexity of the procedures. We managed the instability, when possible, by changing internal modules. In three cases, a cup revision was needed. We did not find failures due the modularity. The two cases of periprosthetic fractures didn’t affect the cup.

Our study presents different limitations. Firstly, all results are presented at mid-term, and longer follow-up is needed to evaluate the long-term survival of these implants. Second, we do not have a control or comparison group and we compared our results with other series presented in the literature with the same material or other trabecular cups. Finally, the time frame of this series is relatively long as patients have been enrolled for 9 years. On the one hand this is justified by the type of procedure (complex acetabular revisions), and on the other by the large series.

## Conclusions

Trabecular titanium revision cups ensured an adaptable system in complex revision cases. No failures caused by the modularity were found in our series. The cups allowed early patient mobilization and weight-bearing thanks to an effective primary stability. These results are encouraging, but further studies are needed to assess long-term survivorship of these implants.

## Data Availability

Data and materials are available on a dedicated dataset, the datasets used and/or analysed during the current study are available from the corresponding author on reasonable request.

## Abbreviations

THA: Total hip arthroplasty
TT: Trabecular titanium
TM: Trabecular metal
HHS: Harris hip score
